# Telomerase Dysfunction in the Tumorigenesis of Genetic Disorders

**DOI:** 10.22088/IJMCM.BUMS.10.1.56

**Published:** 2021-05-22

**Authors:** Maha Mohamed Farid Aql, Seham Abd-El Ghafour Bahget, Naglaa Kholoussi, Ghada Mohamed El Hossiny Abdel-Salam, Haiam Abdel Raouf, Maha Mohamed Eid, Rania El-Bialy Esmail

**Affiliations:** 1 *Faculty of Medicine for Girls, Al Azhar University, Cairo, Egypt.*; 2 *Immunogenetic Department, National Research Centre, Cairo, Egypt.*; 3 *Clinical Genetic Department, National Research Centre, Cairo, Egypt.*; 4 *Cytogenetic Department, National Research Centre, Cairo, Egypt.*

**Keywords:** Telomerase, genetic disorder, neurofibromatosis, chromosomal breakage, overgrowth, oxidative stress

## Abstract

Telomeres are nucleoprotein complexes present at the ends of chromosome to maintain its integrity. Telomere length is maintained by an enzyme called "telomerase". Thus, telomerase activity and telomere length are crucial for the initiation of cancer and tumors survival. Also, oxidative stress will cause DNA, protein, and/or lipid damage, which end with changes in chromosome instability, genetic mutation, and may affect cell growth and lead to cancer. Some genetic diseases such as chromosomal instability syndrome, overgrowth syndrome, and neurofibromatosis make the patients at higher risk for developing different types of cancers. Therefore, we aimed to estimate telomerase activity and oxidative stress in these patients. Blood samples were collected from 31 patients (10 with neurofibromatosis, 11 with chromosomal breakage, and 10 with overgrowth syndrome) and 12 healthy subjects. Blood hTERT mRNA was detected by real time quantitative reverse-transcription PCR (RT-qPCR). All patients were subjected to chromosomal examination and chromosome breakage study using diepoxybutane method. Moreover, serum glutathione (GSH), glutathione-s-transferase (GST) activity and nitric oxide (NO) levels were measured among the control and patients groups. Receiver operating characteristic (ROC) curve was drawn to evaluate the efficiency of telomerase activity as a biomarker for the prediction of cancer occurrence. The relative telomerase activity in neurofibromatosis patients was significantly higher than controls (P = 0.014), while it was non-significantly higher in chromosomal breakage and overgrowth patients (P = 0.424 and 0.129, respectively). NO levels in neurofibromatosis, chromosomal breakage and overgrowth patients significantly increased with respect to control (P = 0.021, 0.002, 0.050, respectively). GSH levels were non-significantly lower in neurofibromatosis and chromosomal breakage patients in comparison with the control group, while it remained unchanged in overgrowth patients. The GST activity was significantly upregulated in neurofibromatosis, chromosomal breakage and overgrowth groups in comparison with the control group (P = 0.001, 0.009, and 0.025, respectively). Chromosomal examination revealed normal karyotype in all four chromosomal breakage patients with positive diepoxybutane test. The results of the present study revealed altered telomerase activity and oxidative stress in the studied genetic disorders. More research studies with a larger number of patients are required to confirm whether this alteration is related to cancer occurrence risk or not.

Telomeres are specialized structures of nucleoprotein complexes existing at chromosome ends that protect these ends (1). Their significance in human diseases is important due to their apparent role in cell aging and carcinogenesis (2). DNA sequence of the telomere loses few base pairs in each cell division. A specialized enzyme called telomerase can compensate this progressive shortening in the telomere length. Telomerase enzyme while absent in most normal somatic cells, is activated in most tumors, germ and immortal cells allowing more proliferation with mutation accumulation risk (3). Telomerase consists of two parts; the first part is telomerase reverse transcriptase (TERT) enzyme which adds telomere (TTAGGG) repeats to the ends of chromosomes, and the second part is telomerase RNA component (TERC) which acts as the template for TERT (4). More than 85% of cancer cells contain genetic aberrations and also over expression of human TERT (hTERT). In the future, hTERT could be used for diagnosis and prognosis of tumor progression risk, and set up an adequate therapy (5).

Reactive oxygen species (ROS) has an important role in tumor development. ROS can be produced either from endogenous sources such as peroxisomes, mitochondria, and inflammatory cells or from exogenous sources such as environmental agents, industrial chemicals, and pharmaceuticals. This ROS resulted from oxidative stress may cause damage to DNA, protein, and/or lipid, and lead therefore to genetic mutations, chromosome instability, cell growth disturbance, and end with cancer (6). Oxidative stress is caused by imbalance between ROS production and their elimination by antioxidants. This imbalance leads to damage of important biomolecules and cells (7). Oxidative damage affects telomere length leading to its shortening. Joao *et al*. (2007) stated that oxidative stress interferes with telomere maintenance through its effect on the activity of telomerase (8). Antioxidants are forming the body’s endogenous defense mechanisms that protect against cell damage by free radicals. Neutralization reactions of free radicals and ROS are catalyzed by several enzymes known as antioxidant enzymes. The most potent of these enzymes is the glutathione system which includes glutathione, glutathione pero-xidases, glutathione reductase, and glutathione S-transferases (9).

Genomic instability has a serious role in cancer via increasing genetic changes responsible for progression of cancer cells (10). Genomic instability can occur due to defect in DNA replication, DNA repair, and chromosome separation. Mutation rate at the nucleotide level does not increase in most cancer cells, meaning that elevated incorporation during replication or a failure to correct damaged or mismatched nucleotides is not a common mechanism of cancer instability (11).

Genomic instability, chromosome aberrations, and predisposition to cancer are the main characteristics of syndromes caused by defect in genes involved in signaling, recognition and/or DNA damage repair, DNA processing, apoptosis, cell cycle regulation and/or maintenance of telomeres. Any defect in one of these genes may cause cancer development and many other clinical defects (12).

Some genetic syndromes such as chromosomal instability syndromes, overgrowth syndromes, and neurofibromatosis are characterized by significantly increased risk of cancer (13-15).

Chromosome instability syndromes are a group of inherited disorders characterized by chromosomal instability and breakage. They may lead to an increased tendency to malignancies especially hematopoietic cancers. They include Fanconi syndrome, ataxia-telengectasia, Bloom syndrome, xeroderma pigmentosum, Cockayne syndrome, and Nijmegen breakage syndrome (16).

Overgrowth syndromes are heterogeneous disorders with any growth feature like the head circumference, weight or height being more than the 97^th^ centile or 2–3 standard deviations (SD) above the average for sex and age. Risk of tumorigenesis is one of the striking features of overgrowth syndromes, especially embryonic cancers. Several neoplasms i.e. leukemia; Wilms tumor, astrocytoma, and neuroblastoma were reported in these patients. Beckwith-Wiedemann syndrome, Weaver syndrome, Proteus syndrome, Sotos syndrome and isolated hemihyperplasia are examples of overgrowth syndromes (17).

Neurofibromatosis is a genetic-inherited disorder. Although, it is an autosomal dominant disorder, but half of the cases are caused by *de novo* mutations and no other family members are recognized as affected. This disorder equally affects males and females. In neurofibromatosis, tumors (neurofibromas) are growing in the nerve tissue. Although these tumors are benign, they may lead to serious damage by compressing nerves and other tissues (18). Neurofibromatosis disorder includes neurofibromatosis type 1 (NF1) that results from mutation in neurofibromin 1 gene located on chromosome 17q11 (19) and neurofibromatosis type 2 (NF2) (also called central neurofibromatosis) due to mutation of the merlin gene also known as neurofibromin 2 that is located on chromosome 22q12. NF2 frequency is lower than NF1 as it accounts for only 10% of all cases of NF (20).

The aim of the current study was to assess the telomerase activity as one of the factors that affect telomere function in some groups of tumorigenic genetic disorders compared to the control, and also evaluate the oxidative stress biomarkers as a factor that can affect telomere function in these genetic disorder groups in comparison to healthy controls. 

## Materials and methods

**Subjects**

Thirty one patients were collected from the outpatient clinics of the Genetics Clinics at the Medical Research Centre of Excellency, National Research Centre (NRC), Egypt. Informed consent from the guardians of patients was included in our study and was documented according to the rules of the Ethics Committee in the National Research Centre and Al-Azhar University. In addition, 12 age and sex matched normal controls were included in this study. Patients were grouped according to their diagnosis into 3 groups: neurofibromatosis, chromosomal breakage and overgrowth syndromes.

Diagnosis of neurofibromatosis patients (10 patients) was based on the presence of 2 or more of the above major criteria: 6 or more café au lait spots, axillary or inguinal freckling, 2 or more cutaneous neurofibromas, 1 plexiform neurofi-broma, an optic glioma, 2 or more iris Lisch nodules, a first-degree relative with NF, or characteristic bony lesions (pseudarthrosis, sphenoid wing hypoplasia) (21).

Eleven patients with chromosomal breakage syndrome were categorized according to the clinical criteria as the following: 5 patients with Nijmegen breakage syndrome were characterized by increased tendency to chromosomal breakage and clinically with microcephaly, growth retardation, and recurrent infections (22). Five patients were diagnosed as ataxia telangiectasia when there were cerebellar symptoms in the form of ataxia, intention tremors, slurred speech, and difficulty in coordination in addition to telangiectasia of the eyes. Furthermore, cerebellar atrophy was evident in the brain magnetic resonance imaging (MRI) (23). One patient with Fanconi anemia showed increased number of breakages by diepoxybutane (DEB), anemia, frequent blood transfusions, and microcephaly (24).

 Ten patients with overgrowth syndromes were diagnosed clinically by increased birth weight, macrocephaly and/or increased bone age (17). This group included 3 patients with Weaver syndrome, 2 patients with Beckwith-Wiedemann syndrome, 2 patients with Sotos syndrome, one patient with hemihypertrophy, one patient with Marshall-Smith syndrome, and one undiagnosed patient.

All patients were subjected to clinical evaluation including medical history, pedigree analysis to three generations taking into consideration consanguinity, and full neurological assessment. Also, ophthalmological examination and brain MRI was performed. 

**Sample collection**

Ten mL of venous blood was collected and distributed into tubes as the following: two ml was delivered under aseptic conditions into a plastic tube containing ethylene diamine tetra acetic acid (EDTA) at concentration of (1.2 mg/mL) for RNA extraction and complete blood count (CBC) using automated cell counter (Cell Dyn 1700, USA). Three mL was collected in lithium heparin vacutainer for chromosomal study, and 5 mL in another sterile EDTA vacutainer and centrifuged (2000 rpm) at 4°C for 20 min and 1 mL of plasma was separated and placed into 1.5 mL tube, and then stored at -20°C till their use for measurement of reduced glutathione (GSH), glutathione-s-transferase (GST), and nitric oxide (NO).

**Chromosomal examination**

Chromosomal examination was performed from peripheral blood lymphocytes for all patients to detect chromosomal breakage (25).

**Diepoxybutane (DEB) method**

Two sets of culture were prepared for each patient. Each set of culture contained Roswell Park Memorial Institute (RPMI) media, phytohema-gglutinin, fetal bovine serum, antibiotics, and L-glutamine. 0.5 mL of patient's blood was added to this media. The diepoxybutane was added 48 h before harvest to one set of culture at final concentration of 0.5 μg/tube. The blood was incubated at 37°C for 72 h. Culture harvest was accomplished using colchicine to stop the division. Then hypotonic solution was added. Finally, fixation was done using methanol: glacial acetic acid 3:1v/v. The number of chromosomal breaks was counted for 50 metaphases for cultures treated with DEB (26).

**Relative Telomerase activity by real time PCR**

Total RNA was extracted from blood samples using an RNA extraction kit (QIAamp, Germany) according to the manufacturer’s instructions, and the extracted total RNA was eluted in 30 µL RNase-free water. cDNA was synthesized using High-Capacity cDNA reverse transcription kit (Applied Biosystems, USA) (27). In brief, 10 µL RNA (1 µg) was reversely transcribed in a 20 µL reaction containing 2 µL 10XRT buffer, 0.8 µL 25X dNTPs (100 mM), 2 µL 10X random hexamer primers, 1 µL RT enzyme MultiScribeTM reverse transcriptase and 4.2 µL nuclease-free water. The reverse transcription was performed on Perkin Elmer 9700 thermocycler (USA) instrument under following condition: 25°C for 10 min, 37°C for 120 min, followed by 85°C for 5 min and hold at 4°C).

**Real time PCR quantification**

To determine *hTERT* mRNA levels, qRT-PCR assays were constructed using TaqMan Gene Expression Assay (Applied Biosystems, USA). The target gene sequence was the catalytic subunit of *hTERT* and the calibrator sample was a healthy control. The reference gene (housekeeping gene) was glyceraldehyde phosphate dehydrogenase (*GAPDH*). A single plex reaction was used in this study. RT-PCR was carried out using TaqMan Universal PCR Master Mix. PCR reaction mix was added to the cDNA samples in a sterile 48-wells PCR plate. One Step PCR instrument (Applied Biosystems, USA) was used with cycling conditions of 50°C for 2 min (reverse transcription), 95°C for 10 min (initial denaturation) followed by 40 cycles of 95°C for 15 s, 60°C for 1 min, and 72°C for 1 min for denaturation, annealing, and extension steps, respectively, using assay primers (Applied Biosystems, USA). Total *hTERT* RNA quantification data were normalized to *GAPDH*, and data were analyzed according to the RQ manager program ABI SDS software (ABI 7900, USA) using RQ formula (2^- ∆∆ct^) (28).

**Colorimetric determination of nitric oxide level**

In acid medium and in the presence of nitrite the formed nitrous acid and the end product are coupled with ethylenediamine. The resulting azo dye has a bright reddish-purple color which can be measured at 540 nm (29). NO level was measured in duplicate using NO assay kit (Biodiagnostic 2000, Egypt).

**Determination of the plasma glutathione**

The method is based on the reduction of (2-nitrobenzoic acid) (DTNB) with GSH to produce a yellow compound. The reduced chromogen is directly proportional to GSH concentration, and its absorbance can be measured at 405 nm (30). GSH of all study subjects was determined in duplicate by GSH reduced kit (Biodiagnostic 2000, Egypt). 

**Estimation of glutathione S-transferase activity**

Total GST activity was estimated by measuring the conjugation of 1-chloro-2,4-dinitrobenene (CDNB) with reduced GSH. The conjugation is accompanied by an increase in absorbance at 340 nm. The increase rate is directly proportional to the GST activity in the sample (31). GST activity was measured in duplicate using GST kit (Biodagnostic 2000, Egypt). 

**Statistical analysis**

Data were statistically analyzed using SPSS version 19.0 software (SPSS Inc., Chicago, Illinois, USA). Nonparametric (Mann-Whitney) test was used to compare between 2 groups. Spearman’s rank correlation was performed to test the correlation between parameters. Data were presented as the mean ± SEM. P value of less than 0.05 was considered statistically significant. Receiver operating characteristic (ROC) curve was constructed for telomerase activity, to evaluate the efficiency of telomerase activity as a biomarker for occurrence of cancer against the controls.

## Results

**Demographic data**

Control group included 12 healthy age and sex matched individuals: 5 males and 7 females. Their ages ranged between 2 and 12 years. Neurofib-romatosis patients comprised 3 males and 7 females. Patients’ age was ranging between 1-14 years (mean = 4.4 ± 1.14 years). Parental consanguinity was encountered in 3/10 families (30%). Similarly, affected siblings were encountered in 4/10 families (40%). Chromosomal Breakage syndrome patients comprised 7 males and 4 females. Parental consanguinity was encountered in all families (100%), the age of patients ranged between 1-14 years (mean = 11.63 ± 1.12 years). Similarly, affected siblings were encountered in 6/11 families (54.5%). Overgrowth patients comprised 7 males and 3 females. Parental consanguinity was encountered in 5/10 families (50%). The age of patients ranged between 1-11 years (mean = 5.4 ± 1.32 years). Similarly, affected siblings were encountered in 2/10 families (20%).

**Cytogenetic findings**

Results of chromosomal examination showed normal karyotype in all examined patients, and positive DEB was observed in four patients from chromosomal breakage group.

**Oxidative stress biomarkers**

**Fig. 1:**
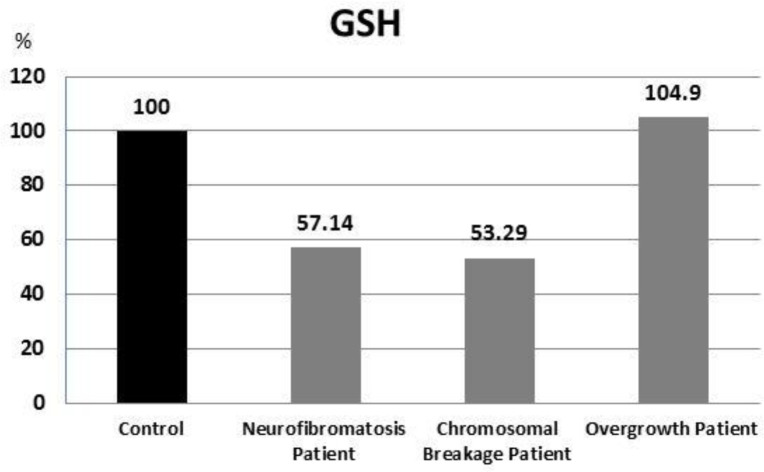
Percent of GSH results in all patients compared with control

GSH showed non-significant lower level in the chromosomal breakage and neurofibromatosis patients in comparison with the control group (P =0.413 and 0.080, respectively). On the other hand, GSH exhibited similar levels in overgrowth patients and control group (Figure 1).

**Fig. 2:**
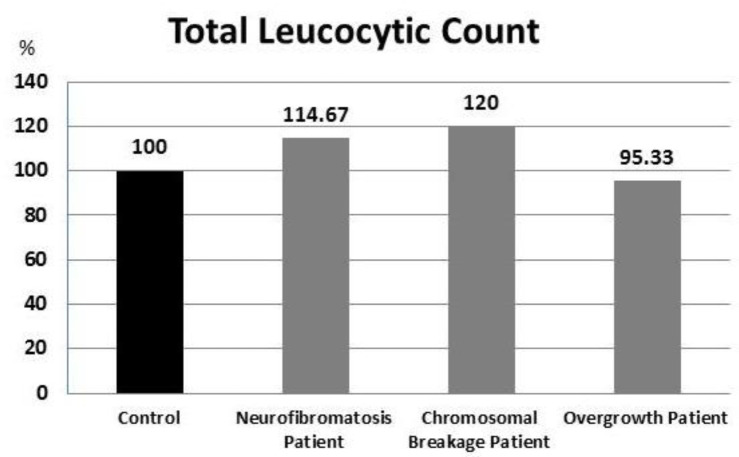
Percent of TLC results in all patients compared with control

TLC results showed no statistically significant differences between all patient groups compared to the control group (Figure 2).

**Fig. 3.**
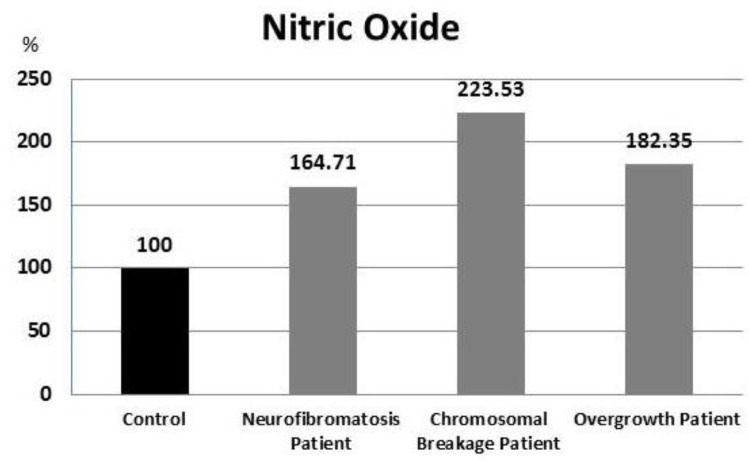
Percent of Nitric Oxide results in all patients in relation to control

NO results showed significantly higher level when compared with control group in neurofib romatosis, overgrowth and chromosomal breakage syndrome patients (P = 0.021, 0.002, and 0.05, respectively) (Figure 3).

**Fig. 4:**
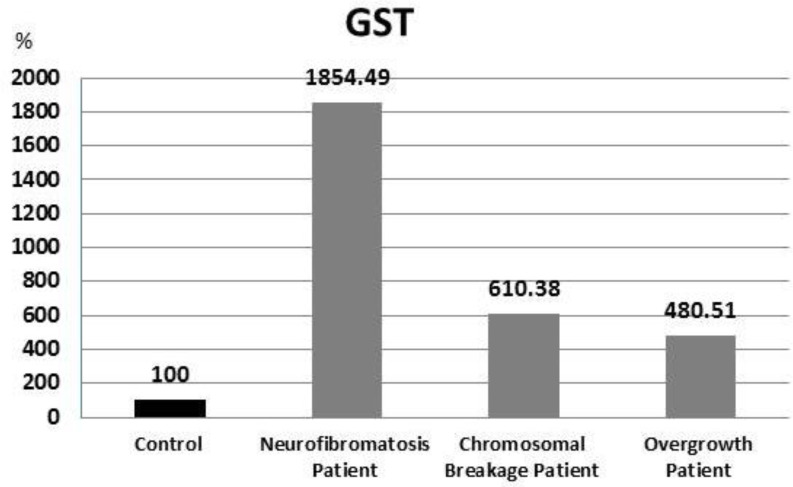
Percent of GST results in all patients in relation to control

GST levels in neurofibromatosis, chromosomal breakage, and overgrowth patients were significantly higher (P = 0.001, 0.009, and 0.025, respectively) in comparison with the control group (Figure 4).

**Gene expression evaluation**

**Fig. 5:**
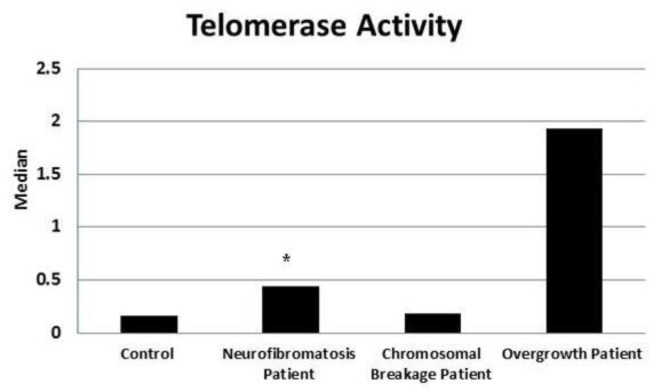
Comparison of telomerase activity in different groups with the control group. *: Significance at P< 0.05 compared with control using Mann-Whitney Test

Telomerase activity assessment is represented in figure 5 significantly higher telomerase activity (2.75 fold, P=0.030) was detected in neurofib-romatosis patients, while a non-significant up regulation was observed in chromosomal breakage (1.13 fold, P = 0.424) and overgrowth (12 fold, P = 0.129) patients in comparison with healthy subjects. 

**Statistical correlations analysis**

Statistical correlations of studied parameters in the neurofibromatosis patients showed that telomerase activity have a non-significant inverse correlation with total leukocytic count, GSH and GST levels (R = 0.377, 0.35, and 0.50, respectively), and non-significant positive correlation with age and NO levels (R = 0.235, and 0.433, respectively) (Table 1)

In the chromosomal breakage patients group, telomerase activity showed non-significant inverse correlation with age, NO, GSH, and GST levels (R = 0.073, 0.336, 0.218, and 0.136, respectively), and non-significant positive correlation with total leukocytic count (R = 0.164) (Table 2).

Telomerase activity in overgrowth patients showed non-significant positive correlation with age, GSH and GST levels (R = 0.241, 0.050, and 0.017 respectively), and a non-significant inverse correlation with total leukocyte count and NO levels (R = 0.160, and 0.033, respectively) (Table 3). 

**ROC curve analysis**

**Fig. 6.**
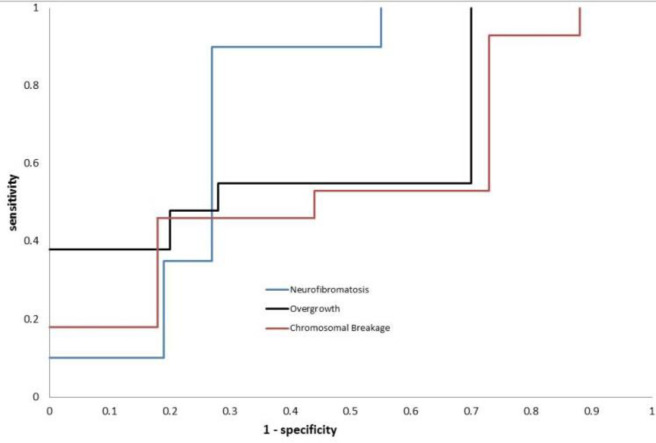
ROC Curve of Telomerase Result in Neurofibromatosis, Overgrowth, and Chromosomal Breakage Patients

ROC curve analysis showed that telomerase activity has an area under the curve (AUC) value of 0.602, a sensitivity of 100%, and a specificity of 33.3% in overgrowth patients while in neurofibromatosis patients the AUC value was 0.725 with a sensitivity of 88.9% and a specificity of 75%. In addition, the ROC curve of telomerase activity in chromosomal breakage group showed an AUC value of 0.904, a specificity of 83.3% and a sensitivity of 45.5% (Figure 6). Prediction results for studied parameters in all groups are summarized in table 4. The specificity of NO was 100% in all studied patient groups and its sensitivity varied between 72.7% and 60%, GSH showed the highest sensitivity in neurofibromatosis patients (90%) and the lowest in overgrowth patients (50%), while its specificity varied between 75% and 58.3%. GST showed a relatively high specificity (75%-91.7 %) and sensitivity (around 80%). 

**Table 1:** Correlations of studied parameters in neurofibromatosis patients (n=10).

	**Age**	**Total leukocytic** **count** x 10^3^/ul	**Telomerase** **activity**	**Nitric** **Oxide** ** (µmol/l)**	**GSH** **(µmol/l)**	**GST** **(U/l)**	**Weight** **(kg)**	**Height** **(cm)**	**OFC** **(cm)**
**Age**		-0.553	0.235	0.403	-0.134	0.084	0.743	0.982**	0.826*
**Total leukocytic** **count** x 10^3^/uL	NS		-0.377	-0.146	-0.219	-0.030	-0.773*	-0.721	-0.773*
**Telomerase **	NS	NS		0.433	-0.350	-0.500	0.577	0.214	0.468
**Nitric Oxide** **(µmol/l)**	NS	NS	NS		-0.467	-0.188	-0.072	0.036	-0.180
**GSH (µmol** **/l** **)**	NS	NS	NS	NS		0.442	-0.126	0.143	0.198
**GST (U/L)**	NS	NS	NS	NS	NS		-0.306	-0.107	-0.090
**Weight (Kg)**	NS	P=0.042	NS	NS	NS	NS		0.775*	0.918**
**Height (cm)**	P=0.000	NS	NS	NS	NS	NS	P=0.041		0.883**
**OFC (cm)**	P=0.022	P=0.042	NS	NS	NS	NS	P=0.004	P=0.008	

P value was calculated using Spearman correlation analysis: ^**^: significant correlation at 0.01 level; ^*^: significant correlation at 0.05 level; NS: non significance; OFC:

occipitofrontal circumference

**Table 2.** Correlations of studied parameters in chromosomal breakage patients (n=11).

	**Age**	**Total** **leukocytic** **count** x 10^3^/uL	**Telomerase **	**Nitric ** **Oxide** **(µmol/l)**	**GSH ** **(µmol/l)**	**GST ** **(U/l)**	**Weight ** **(Kg)**	**Height ** **(cm)**	**OFC ** **(cm)**
**Age**		-0.171	-0.073	0.171	0.098	0.195	0.439	0.439	0.160
**Total leukocytic** **count** x 10^3^/uL	NS		0.164	-0.327	-0.164	0.555	0.055	0.064	0.404
**Telomerase activity**	NS	NS		-0.336	-0.218	-0.136	-0.327	-0.173	0.220
**Nitric Oxide** ** (µmol/l** **)**	NS	NS	NS		-0.100	-0.282	-0.045	0.109	-0.505
**GSH (µmol** **/l** **)**	NS	NS	NS	NS		0.218	-0.118	-0.173	0.257
**GST (U/L)**	NS	NS	NS	NS	NS		0.073	0.064	0.486
**Weight (gm)**	NS	NS	NS	NS	NS	NS		0.918**	0.514
**Height (cm)**	NS	NS	NS	NS	NS	NS	P=0.000		0.514
**OFC (cm)**	NS	NS	NS	NS	NS	NS	NS	NS	

P value was calculated using Spearman correlation analysis: ^**^: significant correlation at 0.01 level; NS: non significance; OFC:

occipitofrontal circumference

**Table 3:** Correlations of studied parameters in overgrowth patients (n=10)

	**Age**	**Total ** **leukocytic** **count** x 10^3^/ul	**Telomerase **	**Nitric ** **Oxide** **(µmol/l)**	**GSH ** **(µmol/l)**	**GST ** **(U/l)**	**Weight ** **(Kg)**	**Height ** **(cm)**	**OFC ** **(cm)**
**Age**		-0.257	0.241	-0.456	-0.218	0.034	0.952**	0.988**	0.855**
**Total leukocytic** **count** x 10^3^/uL	NS		-0.160	0.346	-0.098	-0.268	-0.345	-0.370	-0.588
**Telomerase **	NS	NS		0.033	0.050	0.017	0.017	0.100	-0.183
**Nitric Oxide** **(µmol/l** **)**	NS	NS	NS		-0.444	0.450	-0.477	-0.636	-0.452
**GSH (µmol** **/l** **)**	NS	NS	NS	NS		-0.721*	0.133	0.133	0.100
**GST (U/L)**	NS	NS	NS	NS	P=0.019		-0.167	-0.200	-0.033
**Weight (Kg)**	P=0.000	NS	NS	NS	NS	NS		0.950**	0.917**
**Height (cm)**	P=0.000	NS	NS	NS	NS	NS	P=0.000		0.867**
**OFC (cm)**	P=0.007	NS	NS	NS	NS	NS	P=0.001	P=0.002	

P value was calculated using Spearman correlation analysis: ^**^: significant correlation at 0.01 level; ^*^: significant correlation at 0.05 level; NS: non significance;

OFC: occipitofrontal circumference

**Table 4:** Prediction results for studied parameters in all patients groups

**Parameter**	**Groups**	**Area under the curve**	**Cut off** **value**	**Sensitivity ** **%**	**Specificity ** **%**	**OR**	**PPV**	**NPV**
**Total leukocytic count**	Neurofibromatosis	0.692	8.200	80.0 %	58.3 %	5.600	61.50%	77.80%
	Chromosomal breakage	0.769	10.400	36.4 %	91.7 %	6.286	80.00%	61.10%
	Overgrowth	0.800	10.250	40.0 %	91.7 %	7.333	80.00%	64.70%
**Telomerase activity **	Neurofibromatosis	0.725	0.278	88.9 %	75.0 %	24.000	72.70%	90.00%
	Chromosomal breakage	0.904	0.862	45.5 %	83.3 %	4.167	71.40%	62.50%
	Overgrowth	0.602	0.028	100.0 %	33.3 %	---	52.90%	100.00%
**Nitric ** **Oxide**	Neurofibromatosis	0.598	10.170	70.0 %	100.0 %	5.000	100.00%	80.00%
	Chromosomal breakage	0.864	9.746	72.7 %	100.0 %	5.000	100.00%	80.00%
	Overgrowth	0.606	9.746	60.0 %	100.0 %	4.000	100.00%	75.00%
**GSH**	Neurofibromatosis	0.818	1.478	90.0 %	58.3 %	12.600	64.30%	87.50%
	Chromosomal breakage	0.558	1.331	72.7 %	66.7 %	5.333	66.70%	72.70%
	Overgrowth	0.657	1.860	50.0 %	75.0 %	3.000	62.50%	64.30%
**GST **	Neurofibromatosis	0.742	501.945	80.0 %	91.7 %	44.000	88.90%	84.60%
	Chromosomal breakage	0.529	150.443	81.8 %	75.0 %	13.500	75.00%	81.80%
Overgrowth	0.783	172.938	80.0 %	75.0 %	12.000	72.70%	81.80%

OR: odd ratio; PPV: positive predictive value; NPV: negative predictive value

## Discussion

The present study analyzed telomerase activity, NO, GSH, and GST levels in neurofibromatosis, chromosomal breakage syndr-omes and overgrowth syndromes patients who did not develop cancer until the time of biochemical analyses. The results were compared with normal age and sex matched controls.

Telomerase is highly expressed in most human cancer cells so, it can be considered as a good biomarker for cancer detection. The catalytic domain of telomerase (hTERT) can serve for telomerase activity assessment. Therefore, the amount of hTERT indicates the severity of a cancer (32).

In the present study, there were 2 patients with a family history of neurofibromatosis and cancer development among neurofibromatosis patients. However, no family history of cancer development was recorded in chromosomal breakage syndrome and overgrowth syndrome patients. 

Kiran *et al*. (2008) studied the expression of *TERT* in 23 malignant peripheral nerve sheath tumors (MPNST) using real-time PCR, and found that *TERT* transcripts were detected in all high grades MPNST and in 50% of the low grade MPNST. Also, they reported a significant *TERT* upregulation in high grade MPNST in comparison with low grade MPNST (P = 0.042) (33). 

Venturini *et al*. (2012) analyzed telomerase activity in 49 patients with MPNST (14 with NF1 and 35 sporadic cases). They found that telomerase activity was more frequently expressed in NF1-associated MPNST patients in comparison with sporadic cases (60.0% vs 29.4%) (34). This is in agreement with our findings that indicated a significant increase of the level of telomerase activity in neurofibromatosis patients in comparison with controls, Although telomerase activity levels were not as high as those with overgrowth syndrome. This was suspected because the telomerase activity increases with age as tumor risk associated with neurofibromatosis increases also with age. 

Unexpectedly, in chromosomal breakage and overgrowth patients, the relative telomerase activity was much higher than the controls but did not reach the level of statistical significance. However, the telomerase activity reported in some cases of chromosomal breakage and overgrowth syndromes reached very high levels. It is worth to emphasize that these levels of telomerase activity were higher than that of neurofibromatosis patients. The absence of statistical significance may be due to the low number of analyzed patients. Because telomerase activation is seen in more than 80%–90% of tumors, telomerase has been examined as a biomarker potentially sensitive for screening, early cancer detection, prognosis or in monitoring as an indication of residual disease (35).

Not only the genetic factors, but also many environmental factors may affect telomeres length. Telomeres are highly susceptible to damage due to oxidative stress (36). Different studies have shown that oxidative stress may partially contribute to telomerase activation. An imbalance between oxidants and antioxidants in favor of oxidants is associated with increased levels of telomerase (37).

Oxidative stress may exert a role in several genetic disorders characterized by neurological degeneration cancer and high cancer prevalence, but may also indicate new strategies for treatment of these syndromes (38).

The present study found that NO levels in neurofibromatosis, chromosomal breakage, and overgrowth patients were significantly higher than the controls. In the same context, many studies reported elevation of the serum NO levels in different types of cancer (39). It is well known that the cells of ataxia telangiectasia patients are particularly sensitive to ROS-generating agents (e.g., hydrogen peroxide and NO) resulting in oxidative stress and oxidative damage to important biomacromolecules and cell structures (40).

The current study showed that GSH levels in neurofibromatosis and chromosomal breakage patients were non-significantly lower than the controls and nearly equally to controls in overgrowth patients. This finding is in agreement with that observed by Palanisamy *et al*. (2009) who analyzed GSH levels in 100 subjects (50 newly diagnosed gastric cancer patients and 50 healthy control subjects) and found a statistically significant decrease in the levels of plasma GSH in gastric cancer patients with respect to healthy control subjects (41).

In this study, we observed that in all patient groups, GST activity was significantly higher than controls. In the same direction, Edita *et al*. (2016) observed a statistically significant down regulation of GSH levels in colorectal cancer patients in comparison with healthy volunteers (42).

From all the above results including ROC curve analysis, it may be concluded that telomerase activity and oxidative stress could be important markers to predict the occurrence of cancer. This could be confirmed by further studies on telomerase activity, oxidative stress, and cancer correlation in larger number of patients and long-time patient follow up.

## Conflict of interest

Authors declare no conflict of interest.
